# The Application of a Flowable Composite as a Method for Donor Site Protection After Free Gingival Graft: A Comparative Analysis of Four Techniques

**DOI:** 10.3390/jcm14176009

**Published:** 2025-08-25

**Authors:** Tomasz Jankowski, Agnieszka Jankowska, Wojciech Kazimierczak, Joanna Janiszewska-Olszowska

**Affiliations:** 1 Private Practice Dental Clinic Jankowscy, Czerwonego Krzyża 24, 68-200 Żary, Poland; agnieszkajankowska2301@gmail.com; 2 Kazimierczak Private Medical Practice, Dworcowa 13/u6a, 85-009 Bydgoszcz, Poland; w.kazimierczak@cm.umk.pl; 3 Department of Radiology and Diagnostic Imaging, Collegium Medicum, Nicolaus Copernicus University in Toruń, Jagiellońska 13-15, 85-067 Bydgoszcz, Poland; 4 Department of Interdisciplinary Dentistry, Pomeranian Medical University in Szczecin, 71-111 Szczecin, Poland; joanna.janiszewska.olszowska@pum.edu.pl

**Keywords:** free gingival graft, palatal wound, donor site, wound healing, pain management, flowable composite

## Abstract

**Background/Objectives:** Soft tissues are essential for maintaining the function and long-term success of dental implants. In many cases, implant placement necessitates soft tissue augmentation procedures such as free gingival grafts (FGGs) or connective tissue grafts (CTGs) to restore lost gingival architecture. Nevertheless, a significant challenge associated with FGG and CTG is postoperative pain, largely due to morbidity at the palatal donor site. To address this issue, various approaches have been proposed to reduce patient discomfort and promote improved wound healing at the donor site. This study aimed to compare the effectiveness of four different methods for protecting the palatal donor site following free gingival graft harvesting. **Methods**: A total of 76 patients undergoing implant therapy with an indication for free gingival grafting were selected and divided into four groups based on the method used to protect the palatal donor site: an absorbable gelatin sponge secured with sutures (GS); an absorbable gelatin sponge with sutures and cyanoacrylate tissue adhesive (GS+CTA); oxidized regenerated cellulose combined with cyanoacrylate tissue adhesive (ORC+CTA); and an absorbable gelatin sponge covered with a flowable resin composite and stabilized with sutures (GS+FRC). The effectiveness of each method was evaluated in terms of postoperative pain, bleeding, and wound healing. **Results**: Although the differences in pain intensity among the groups were not statistically significant throughout the observation period (*p* > 0.05), the GS+FRC group consistently exhibited the lowest mean pain scores. No statistically significant differences were observed between the groups regarding the incidence of secondary bleeding. The highest mean wound healing rate was recorded in the GS+FRC group (75.95 ± 18.75%), whereas the ORC+CTA group demonstrated the lowest rate (43.66 ± 25.74%). **Conclusions**: The use of an absorbable gelatin sponge covered with a flowable resin composite and secured with sutures, despite the presented limitations, appears to be a promising approach for palatal wound protection. While this group consistently demonstrated the lowest mean pain scores, differences in pain intensity among the groups were not statistically significant. Nonetheless, it achieved the most favorable outcomes in terms of wound epithelialization.

## 1. Introduction

Dental implant therapy has become a widely accepted and increasingly common method for replacing missing teeth in dental practice [1]. According to the literature, soft tissues are crucial for maintaining the proper function and overall health of dental implants [2,3,4,5]. In 1960, Clifford Ochsenbein highlighted that the attached gingiva is uniquely structured to meet functional demands, unlike the alveolar mucosa, which is thinner and less securely connected, making it unsuitable for chewing purposes [6]. A minimum of 2 mm of keratinized mucosa surrounding dental implants has been positively associated with improved peri-implant tissue stability, including reduced plaque accumulation, lower inflammation, and the preservation of crestal bone levels [7].

The absence of teeth contributes to the reduction in gingival tissue, primarily due to the lack of mechanical stimulation, which accelerates soft tissue atrophy and leads to alveolar bone resorption [8,9]. As a result, delayed implant placement frequently requires additional soft tissue augmentation procedures, such as free gingival grafts (FGGs) or connective tissue grafts (CTGs), to reconstruct the lost gingival architecture [10]. In contrast, immediate implant placement may reduce or eliminate the need for such interventions by preserving soft tissue volume and promoting more favorable esthetic outcomes, particularly in the anterior maxilla [11].

A major concern associated with FGG and CTG procedures is postoperative pain, primarily related to morbidity at the palatal donor site [12]. Consequently, several strategies have been proposed to minimize patient discomfort and enhance wound healing at the donor area. Reported methods include the use of an absorbable gelatin sponge [13,14,15], a gelatin sponge combined with cyanoacrylate [16], platelet-rich fibrin (PRF) [17,18,19,20], oxidized regenerated cellulose [21], collagen membranes [22], and acrylic plates [23,24]. A more recent approach involves the application of a flowable resin composite coating over a collagen sponge, stabilized with sutures, which has shown promising results in pain control [25]. In addition to mechanical dressings, physical therapies such as photobiomodulation (PBM) [26,27,28], non-thermal atmospheric pressure plasma (NAPP) [29], and microcurrent electrical stimulation [30] have also been explored for their potential benefits in improving patient comfort and accelerating tissue repair.

Although numerous studies have evaluated individual methods of palatal wound protection, direct comparisons among multiple techniques remain limited. The present study aimed to compare four different methods for protecting the palatal donor site following free gingival graft harvesting:

1. An absorbable gelatin sponge with sutures (GS).
2. An absorbable gelatin sponge with sutures and cyanoacrylate tissue adhesive (GS+CTA).
3. Oxidized regenerated cellulose with cyanoacrylate tissue adhesive (ORC+CTA).
4. An absorbable gelatin sponge covered by a flowable resin composite and secured with sutures (GS+FRC).

These methods were assessed in terms of postoperative pain, bleeding, and wound healing.

## 2. Materials and Methods

This research received approval on 18 September 2023 from the ethics committee of the Regional Medical Chamber in Zielona Góra, Poland (resolution number 02/174/2023). This study adhered to the ethical guidelines set forth in the Helsinki Declaration for medical research involving human subjects, as revised at the 64th WMA General Assembly in Fortaleza, Brazil, October 2013. This investigation is a prospective, randomized, controlled clinical trial utilizing a parallel-arm design, conducted at the Private Practice Dental Clinic Jankowscy in Żary, from October 2023 to February 2025.

### 2.1. Study Population

A total of 76 patients undergoing implant therapy with an indication for free gingival grafting were selected based on the following inclusion criteria:

- Aged over 18 years;
- No history of prior palatal harvesting;
- Less than 2 mm of keratinized mucosa in the vicinity of the implant;
- Generally healthy or with well-managed systemic conditions (e.g., hypertension or hypothyroidism) that do not adversely affect healing or bleeding.

The exclusion criteria were as follows:

- Pregnancy or lactation;
- History of alcohol or drug abuse;
- Receipt of chemotherapy, corticosteroids, or immunosuppressants within the past 6 months;
- Coagulation disorders or current use of anticoagulant therapy;
- Poorly controlled systemic conditions (e.g., hypertension or type 2 diabetes mellitus) that may impair healing or bleeding;
- Active oral infections (bacterial, viral, or fungal);
- History of radiation therapy to the head and neck region.

### 2.2. Surgical Procedures

Immediately before surgery, the ultrasonic scaling of the mouth was performed, followed by rinsing with a 0.1% chlorhexidine digluconate mouthwash for 60 s. All surgical procedures were conducted by an oral surgery specialist (TJ) following the Sullivan and Atkins technique [31], and they were performed under local anesthesia using articaine with adrenaline (Dentocaine 40 mg/mL + 0.01 mg/mL; Laboratorios Inibsa, S.A.; Llicà de Vall, Spain).

When the recipient site was prepared, the harvesting of the free graft from the palate began. The graft was approximately 2 mm thick, and its size was adapted to the specific needs of the recipient area. A sterile template from the paper was created to match the dimensions of the FGG. The graft donor area comprised the region between the canine and second molar. The horizontal cut adjacent to the teeth was made roughly 1.5–2 mm from the tooth margin. After harvesting the graft, bleeding was controlled by applying pressure with sterile gauze soaked in saline solution.

Following the application of the graft to the recipient site, the palatal wound was measured using a periodontal probe (mm^2^) and defined as the primary wound area (PWA).

### 2.3. Donor Site Management

**Figure 1:**
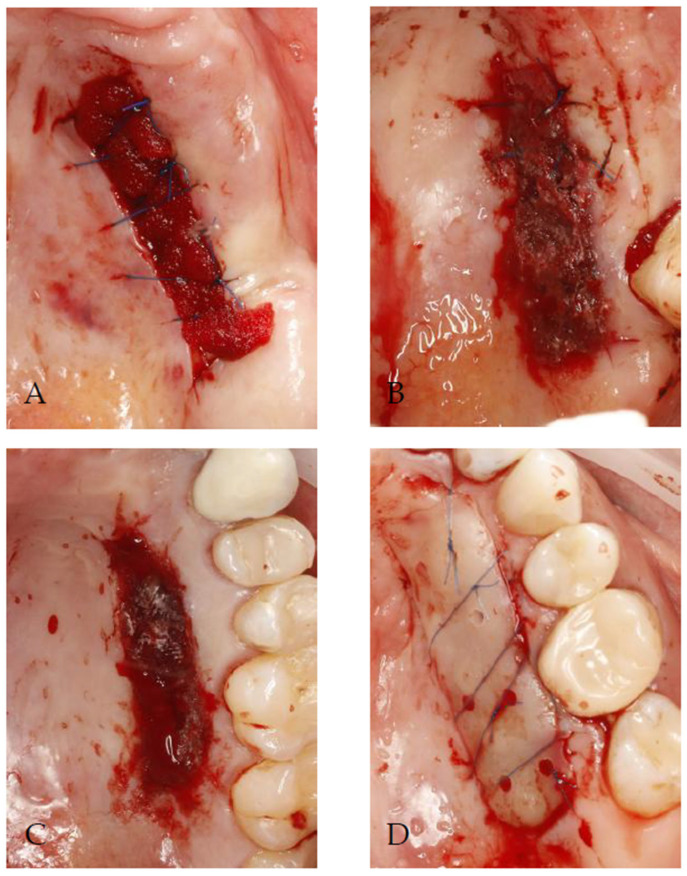
Methods of palatal wound protection following free gingival grafting used in present study: (**A**) absorbable gelatin sponge stabilized by sutures; (**B**) absorbable gelatin sponge stabilized by sutures and sealed with cyanoacrylate tissue adhesive as secondary layer; (**C**) oxidized regenerated cellulose sealed with cyanoacrylate tissue adhesive as second layer; (**D**) absorbable gelatin sponge covered by flowable resin composite and secured with sutures.

The patients were randomly allocated into one of the four groups by drawing lots (Figure 1).

**Figure 2:**
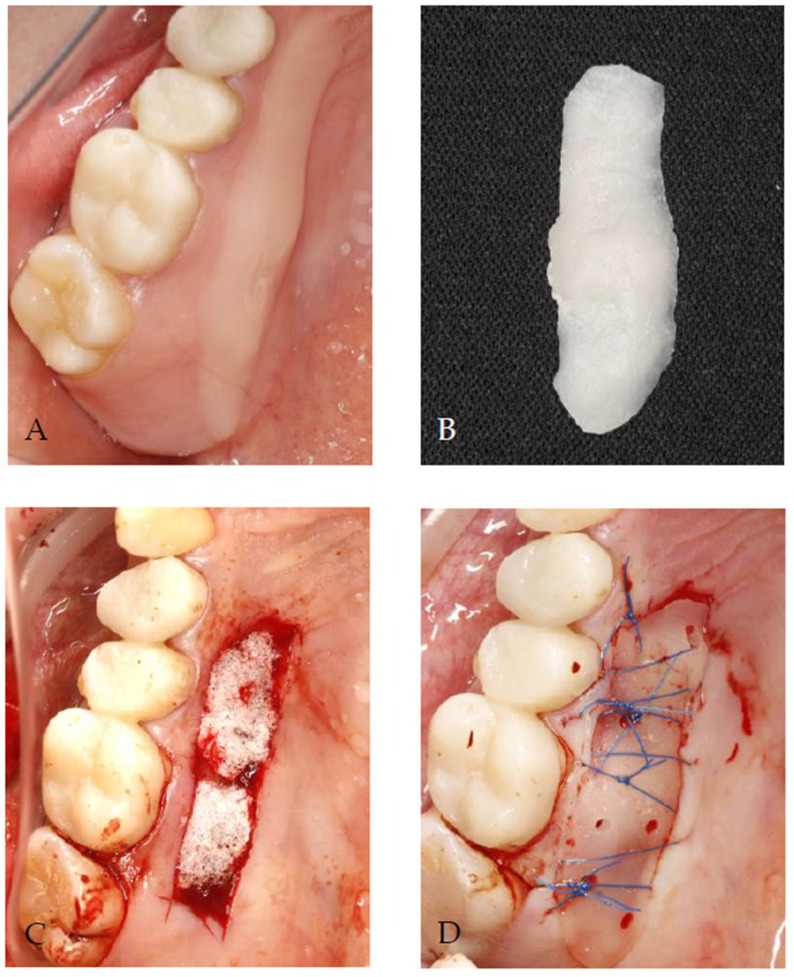
The procedure performed in test group III: (**A**) prior to the harvesting of the free gingival graft, the donor site was covered with a flowable composite material, which was subsequently light-cured; (**B**) the removal of the composite plate from the palate, the smoothing of sharp edges, and disinfection with 0.1% chlorhexidine digluconate; (**C**) after the graft was removed, a gelatin sponge was placed at the donor site; (**D**) the suturing of the composite plate using mattress sutures.

(1) GS—control group (n = 18):
  - Absorbable gelatin sponge (Spongostan Dental, Ethicon, Cincinnati, OH, USA) stabilized by 5/0 nylon mattress sutures (Seralon, Serag-Wiessner, Naila, Germany).
(2) GS+CTA—test group I (n = 20):
  - Absorbable gelatin sponge stabilized by 5/0 nylon mattress sutures.
  - Cyanoacrylate tissue adhesive (PeriAcryl^®^90, GluStitch, Delta, BC, Canada) as a second layer.
(3) ORC+CTA—test group II (n = 19):
  - Oxidized regenerated cellulose (BloodSTOP^®^iX, LifeScience PLUS Inc., Mountain View, CA, USA).
  - Cyanoacrylate tissue adhesive as a second layer.
(4) GS+FRC—test group III (n = 19):
  - Before the surgical procedure, the plate made of a flowable resin composite, in which the perforations were made, was prepared and disinfected with 0.1% chlorhexidine digluconate.
  - An absorbable gelatin sponge was inserted directly on the wound.
  - The composite plate was positioned with 5/0 nylon simple sutures passing through the perforations and stabilized with 5/0 nylon mattress sutures.
  - Figure 2 presents the complete procedure.

### 2.4. Postoperative Care

- To rinse, 0.1% chlorhexidine digluconate mouthwash (Eludril Classic, Pierre Fabre S.A., Paris, France) should be used twice daily for 14 days.
- Preoperative antibiotic prophylaxis involves taking 2000 mg of amoxicillin one hour before surgery, followed by a postoperative dose of 500 mg every eight hours for six days. For patients allergic to penicillin, 600 mg of clindamycin is administered one hour before surgery, followed by a postoperative dose of 300 mg three times a day for six days.
- Painkillers: Both 50 mg of ketoprofen and 500 mg of paracetamol are taken three times a day for three days. For the subsequent days, participants were instructed to use these medications as needed.
- Seven days after surgery, the sutures in the donor area were removed in the control group (GS) and test group I (GS+CTA). In test group III (GS+FRC), the composite plate was removed along with the sutures at the same time.
- In all groups, the sutures at the recipient site were taken out after two weeks.

### 2.5. Outcome Measurements

After surgery, study participants received a 14-day questionnaire evaluating two postoperative parameters: pain (P) and secondary bleeding (SB). SB was indicated in the questionnaire by marking a “+” for presence or a “−” for the absence of bleeding. Pain was recorded using the Visual Analog Scale (VAS) [32], where 0 represents “no pain”, and 10 signifies “the worst pain imaginable.”.

**Figure 3:**
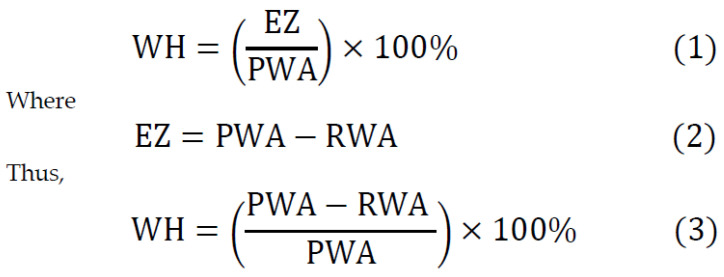
(1) Wound healing percentage (WH), defined as the epithelialization zone (EZ) divided by the present wound area (PWA) and expressed as a percentage; (2) The epithelialization zone (EZ), determined by subtracting the remaining wound area (RWA) from the primary wound area (PWA); (3) Formula (3), representing the extension of Formula (1) by incorporating Formula (2).

Fourteen days after surgery, epithelialization was evaluated using gauze soaked in 3% hydrogen peroxide (H_2_O_2_) to observe bubbling in the wound. The area without epithelial coverage (mm^2^), referred to as the remaining wound area (RWA), was identified by the presence of bubbles and measured with a periodontal probe. The epithelialization zone (EZ) was determined by subtracting the remaining wound area (RWA) from the primary wound area (PWA). The wound healing percentage (WH) was calculated using the formula presented in Figure 3.

### 2.6. Statistical Analysis

The mean, standard deviation, median, quartiles, and range of quantitative variables were calculated. For qualitative variables, absolute and relative frequencies (N and %) were reported. Chi-squared test (with Yates correction for 2 × 2 tables) or Fisher’s exact test (in case of low expected values) was used for comparisons of qualitative variables between groups. The Mann–Whitney test was used for comparisons of quantitative variables between two groups, while the Kruskal–Wallis test (followed by post hoc Dunn test) was used for three or more groups. The significance level was set to 0,05. All the analyses were conducted in R software, version 4.5.0 [33].

### 2.7. Use of Generative AI Tools

In the process of manuscript preparation, ChatGPT (GPT-5, OpenAI) was employed to support the organization, formatting, and verification of references.

## 3. Results

This study included 46 females (60.53%) and 30 males (39.47%), with a mean age of 53.93 years (SD = 11.23). Fourteen patients (18.42%) were smokers. Table 1 presents the demographic and clinical characteristics of the participants across the four groups, categorized by sex, age, graft harvest site, and smoking status. There were no statistically significant differences in the distribution of sex, age, or smoking status between the groups. However, a significant difference was observed in the distribution of the graft harvest site between the groups (*p* = 0.004).

There were no statistically significant differences between smokers and non-smokers regarding any of the evaluated parameters.

Although differences in pain intensity among the groups were not statistically significant throughout the observation period (*p* > 0.05), group IV (GS+FRC) consistently demonstrated the lowest mean pain scores, both during the initial 7-day period and over the entire 14-day assessment (Table 2). During the first 7 days, the mean pain intensity was significantly higher in females than in males; however, no sex-based differences were observed between days 8 and 14.

No statistically significant differences were found between groups or genders with respect to the incidence of secondary bleeding.

A statistically significant difference in wound healing percentage was observed among the groups on day 14 (*p* = 0.003). The GS+FRC group showed the highest mean wound healing rate (75.95 ± 18.75%), while the ORC+CTA group had the lowest (43.66 ± 25.74%). The data regarding wound healing are presented in Table 3.

## 4. Discussion

Free gingival graft (FGG) procedures are commonly associated with significant postoperative discomfort, particularly due to pain at the palatal donor site, which heals by secondary intention and is therefore more sensitive and exposed [34,35,36,37]. In the current investigation, four methods were compared in terms of both subjective and objective parameters. An absorbable gelatin sponge is frequently used as a control group in several investigations; therefore, this method appears to be the standard approach for protecting the palatal donor site [16,38,39]. In a study by Meza-Mauricio et al., a new approach was proposed using flowable resin to reduce postoperative pain in the palatal region following free gingival graft harvesting [25].

In the present investigation, no statistically significant differences were observed between smokers and non-smokers regarding any of the evaluated parameters. However, previous studies have reported contrasting findings. In a study by Silva et al., immediate bleeding was significantly more prevalent in non-smokers (75%) compared to smokers (30%). Furthermore, at 15 days postoperatively, the complete epithelialization of the palatal donor site was observed in 92% of non-smokers versus only 20% of smokers [40]. Similarly, Tawfik et al. stated that non-smokers exhibited better healing outcomes, including reduced discomfort and faster epithelialization, compared to smokers [41].

### 4.1. Pain Perception

While intergroup differences in pain perception were not statistically significant, the group receiving the flowable resin composite plate reported the lowest mean pain scores, suggesting a potential clinical benefit that warrants further investigation. According to Meza-Mauricio et al. [25], the application of flowable resin over a collagen sponge, stabilized with sutures, resulted in a statistically significant reduction in pain scores. For comparison, in the present study, day 1 pain scores were 3.83 in the control group and 3.63 in the flowable composite group. In contrast, Meza-Mauricio et al. reported day 1 scores of 4.94 in the control group and 1.63 in the intervention group, indicating a more pronounced analgesic effect in their findings [25]. Comparing day 7 scores, Meza-Mauricio et al. found scores of 1.44 in the control group and 0.26 in the intervention group, whereas in the present study, the corresponding values were 1.94 and 1.53, respectively.

In their study, Meza-Mauricio et al. applied a flowable composite to the borders of the collagen sponge to achieve mechanical retention on the palatal surfaces of the upper teeth. However, clinical observations by the first author of the present publication revealed that this composite frequently detached from the palatal surfaces, leading to patient discomfort and dissatisfaction. Additionally, during polymerization, liquid dental composites can release toxic monomers such as Bis-GMA, HEMA, and TEGDMA, which have been shown to exhibit cytotoxic and allergenic effects on oral tissues [42]. Matsuura et al. [43] recently demonstrated that the degree of composite toxicity may depend on factors such as material composition, curing method, and surface treatment. Although their study did not directly assess cellular responses, the results underscore the need for further investigation into the biocompatibility of materials, particularly those in prolonged contact with soft oral tissues [43].

To overcome this limitation, the first author proposed the direct suturing of a prefabricated composite plate to the palatal soft tissues, which improved mechanical stability and enhanced patient comfort. Meanwhile, Laguna-Martos et al. described a similar approach called the “Patchwork Technique” [44]. In the Patchwork Technique, however, a collagen sponge—rather than a gelatin sponge—was sutured directly onto the wound bed after graft harvesting. Subsequently, a flowable composite material was applied over the collagen sponge, without the prior preparation of a composite plate. Laguna-Martos et al. [44] concluded that this method reduces postoperative pain and promotes wound healing; nevertheless, since their publication was a case report, it does not provide strong scientific evidence.

Rossmann and Rees [21] stated that oxidized regenerated cellulose did not provide any advantage in postoperative pain reduction compared to an absorbable gelatin sponge or sterile gauze compression. Numerous studies in the literature have investigated the application of platelet-rich fibrin (PRF) to the palatal donor site following free gingival graft (FGG) harvesting. However, the results are variable; while some studies report that PRF provides a significant benefit in reducing postoperative pain [17,18,45,46], others do not demonstrate such an effect [19,20,22,47]. Several studies have also demonstrated that photobiomodulation does not result in a significant improvement in pain perception [27,28,48,49].

### 4.2. Wound Healing

In terms of wound healing, the present study revealed that the group treated with an absorbable gelatin sponge covered by a flowable resin composite and secured with sutures (GS+FRC) exhibited the highest healing rate. One possible explanation for the superior wound healing observed in the GS+FRC group is the elevated mechanical stability provided by the prefabricated composite plate. Unlike traditional dressing methods, which may shift or be removed, the composite plate was stably secured with sutures, minimizing micromovements and mechanical trauma at the donor site. This mechanical shielding may have allowed for uninterrupted epithelial migration and protected the early clot from distortion.

The composite material also has other applications in implantology regarding wound healing; for example, it is commonly used for fabricating a customized healing abutment on a standard abutment during immediate implantation. This approach supports soft tissue healing, as confirmed by Menchini-Fabris et al. [50], whose findings indicate that a protocol involving immediate implant placement combined with a customized healing abutment made of PEEK and composite resin can be an effective treatment strategy in periodontally compromised sockets. This technique demonstrated promising results in preserving alveolar ridge width and maintaining an adequate buccal zone of keratinized mucosa. Additional studies support these benefits: a randomized clinical trial comparing customized versus standard healing abutments in immediate implants with bone grafting found significantly better papilla preservation and reduced mesial bone loss in the customized group [51]. Furthermore, a recent review showed that a resin composite applied to PEEK or titanium bases is a reliable material for customizing healing abutments, allowing for the accurate shaping of the peri-implant emergence profile while maintaining good mechanical and biological properties [52].

In contrast, the group treated with oxidized regenerated cellulose and cyanoacrylate tissue adhesive as a secondary layer (ORC+CTA) showed the lowest healing rate. Conversely, Rossmann and Rees [21] reported opposite findings, demonstrating that oxidized regenerated cellulose (ORC) promoted superior palatal wound healing compared to an absorbable gelatin sponge. To this date, no study has investigated wound healing outcomes following the use of a flowable resin composite as a protective layer. The literature demonstrates that platelet-rich fibrin [13,15,18,45,47], photobiomodulation [27,28,48,49], and ozone [53] accelerate wound epithelialization.

### 4.3. Study Limitations and Risk of Bias

Although this study provides new perspectives into palatal wound protection following FGG harvesting, several limitations should be noted. First, the sample size, while adequately powered for detecting differences in wound healing, may limit the external validity of the findings. Second, the 14-day follow-up period restricts the ability to evaluate long-term outcomes such as complete wound healing or late complications. However, patients were routinely informed—as is standard for such procedures—that they should report any concerning symptoms. None of the participants reported complications beyond the 14-day observation period. Furthermore, since FGG harvesting is one of the intermediate, but not final, stages of implant therapy, all patients returned for follow-up treatment no later than three months after the procedure.

Third, the absence of blinding introduces a potential for bias, especially in subjective outcomes such as pain perception. Participants’ awareness of receiving a more advanced or protective treatment such as the prefabricated flowable composite plate may have influenced their self-reported pain scores due to placebo effects or expectancy bias.

Lastly, while randomization was employed to minimize selection bias, the unequal distribution of graft harvest sites across groups (*p* = 0.004) could have subtly affected healing outcomes due to anatomical or vascular differences in palatal regions. Additionally, all procedures were performed by the same right-handed surgeon (T.J.), which ensured procedural consistency but also introduced potential operator bias.

Future research should aim to address these limitations by incorporating the blinding of participants and outcome assessors, extending follow-up durations beyond the early healing phase, and using objective digital methods to quantify wound healing and pain responses.

## 5. Conclusions

(1) The use of an absorbable gelatin sponge covered with a flowable resin composite and secured with sutures, despite the presented limitations, appears to be a promising method for palatal wound protection. Although this group consistently demonstrated the lowest mean pain scores, differences in pain intensity among the groups were not statistically significant. Nevertheless, it showed the most favorable outcomes in terms of wound epithelialization.
(2) Suturing a prefabricated composite plate to the palatal soft tissues appears to be a viable approach, as the direct polymerization of a composite material on the palatal wound may exert cytotoxic and allergenic effects on the surrounding soft tissue.
(3) No single method proved superior in preventing secondary bleeding among the evaluated approaches.
(4) Further large-scale research is warranted to investigate the use of a flowable composite for palatal wound protection, particularly given the limited evidence currently available in this area. Additionally, multicenter trials and histological assessments should be considered to validate and expand upon these findings.

## Figures and Tables

**Table 1:** Baseline characteristics of patients by treatment group.

Parameter		GS (N = 18)	GS+CTA (N = 20)	ORC+CTA (N = 19)	GS+FRC (N = 19)	Total (N = 76)	*p*
Sex	Female	9 (50.00%)	14 (70.00%)	10 (52.63%)	13 (68.42%)	46 (60.53%)	*p* = 0.461
	Male	9 (50.00%)	6 (30.00%)	9 (47.37%)	6 (31.58%)	30 (39.47%)	
Age	Mean (SD)	55.44 (10.07)	55.85 (9.23)	50.42 (15.95)	54 (8.14)	53.93 (11.23)	*p* = 0.734
	Median (quartiles)	59.5 (49.25–63.75)	54 (49.75–64)	49 (40.5–63.5)	53 (50.5–61)	53.5 (48–63)	
	Range	36–67	38–72	20–72	36–66	20–72	
	n	18	20	19	19	76	
Side	Left	4 (22.22%)	6 (30.00%)	14 (73.68%)	11 (57.89%)	35 (46.05%)	*p* = 0.004 *
	Right	14 (77.78%)	14 (70.00%)	5 (26.32%)	8 (42.11%)	41 (53.95%)	
Smoking	Smokers	2 (11.11%)	4 (20.00%)	5 (26.32%)	3 (15.79%)	14 (18.42%)	*p* = 0.709
	Non-smokers	16 (88.89%)	16 (80.00%)	14 (73.68%)	16 (84.21%)	62 (81.58%)	

*p*—Qualitative variables: chi-squared or Fisher’s exact test. Quantitative variables: Kruskal–Wallis test. * statistically significant (*p* < 0.05). GS—an absorbable gelatin sponge with sutures; GS+CTA—an absorbable gelatin sponge with sutures and cyanoacrylate tissue adhesive; ORC+CTA—oxidized regenerated cellulose with cyanoacrylate tissue adhesive. GS+FRC—an absorbable gelatin sponge covered by a flowable resin composite and secured with sutures.

**Table 2:** Peak and average pain intensity scores (VAS) over 14 days by treatment group.

Parameter	Method	N	Mean	SD	Median	Min	Max	Q1	Q3	*p*
Peak pain intensity	GS	18	4.33	2.59	5	0	9	2	6	*p* = 0.608
	GS+CTA	20	4.45	2.82	5	1	10	2	6	
	ORC+CTA	19	5.21	2.72	5	0	9	4	7	
	GS+FRC	19	4.11	2.16	3	1	8	3	5.5	
Average pain intensity Days 1–14	GS	18	1.75	1.45	1.39	0	4.5	0.45	3.11	*p* = 0.44
	GS+CTA	20	2.01	1.62	1.86	0.07	6.64	0.64	2.95	
	ORC+CTA	19	2.08	1.49	1.79	0	4.29	1	3.5	
	GS+FRC	19	1.35	0.97	1.29	0.07	3	0.46	2.14	
Average pain intensity Days 1–7	GS	18	2.81	2.18	2.5	0	6.43	0.89	4.57	*p* = 0.367
	GS+CTA	20	3.09	2.46	3.43	0.14	9.14	0.86	4.18	
	ORC+CTA	19	3.35	2.23	3	0	7.14	1.86	5.07	
	GS+FRC	19	2.09	1.46	1.86	0.14	4.43	0.71	3.14	
Average pain intensity Days 8–14	GS	18	0.69	0.94	0.21	0	3.29	0	0.96	*p* = 0.726
	GS+CTA	20	0.93	1.05	0.64	0	4.14	0.21	1.32	
	ORC+CTA	19	0.8	0.96	0.29	0	2.86	0	1.71	
	GS+FRC	19	0.62	0.79	0.29	0	3	0	0.93	

*p*—Kruskal–Wallis test; SD—standard deviation; Q1—lower quartile; Q3—upper quartile.

**Table 3:** Wound healing percentage on day 14 by treatment group.

Method	N	Wound Healing [%]							*p*
		Mean	SD	Median	Min	Max	Q1	Q3	
GS	18	63.05	19.72	65.71	28.57	92	57.14	76.45	*p* = 0.003 *
GS+CTA	20	50.36	35.84	43.65	4.76	100	17.94	86.81	
ORC+CTA	19	43.66	25.74	35.71	9.09	100	26.39	56.16	
GS+FRC	19	75.95	18.75	76.19	42.18	100	61.30	92.54	

*p*—Kruskal–Wallis test + post hoc analysis (Dunn test); SD—standard deviation; Q1—lower quartile; Q3—upper quartile. * statistically significant (*p* < 0.05).

## Data Availability

All the raw data are available from the corresponding authors upon reasonable request.
